# The value of second-line anti-HER2 therapy in metastatic HER-2 positive patients: a cost-effectiveness analysis in China

**DOI:** 10.3389/fphar.2024.1382120

**Published:** 2024-07-12

**Authors:** Lu Li, Shilei Yang, Fengqi Fang, Li Tian, Ying He, Jia Li, Yanwei Chen, Deshi Dong

**Affiliations:** ^1^ Department of Pharmacy, First Affiliated Hospital of Dalian Medical University, Dalian, China; ^2^ Department of Oncology, First Affiliated Hospital of Dalian Medical University, Dalian, China

**Keywords:** HER-2-positive metastatic breast cancer, network meta-analysis, cost-effectiveness analysis, pyrotinib plus capecitabine, T-DM1, T-DXd

## Abstract

**Background:**

Breast cancer (BC) is one of the most common cancers worldwide. The inevitability of drug resistance to initial anti-HER-2 therapy necessitates the emergence of second-line anti-HER-2 drugs which exhibit a promising outlook. Consequently, it is imperative to appraise their efficacy through network meta-analysis and ascertain their comparative cost-effectiveness.

**Methods:**

The data used in our analysis were acquired from patients enrolled in the EMILIA, DESTINY-Breast03, and PHOEBE phase III randomized clinical trials. A partitioned survival model was used for patients diagnosed with HER-2-positive metastatic Breast cancer. The model was crafted with a time horizon of 10 years, operating on a 21-day cycle and incorporating a 5% discount rate for both costs and outcomes. The willingness-to-pay threshold was set at $36,058.06 per quality-adjusted life year (QALY). The impact of parameter uncertainty on the findings was assessed using a one-way deterministic sensitivity analysis and probability sensitivity analysis.

**Findings:**

Within the model encompassing 1782 patients, the utilization of pyrotinib plus capecitabine (PC) treatment yielded an additional 0.70 QALY in comparison to T-DM1, resulting in an incremental cost-effectiveness ratio (ICER) of $31,121.53 per QALY gained. Similarly, the administration of T-DXd treatment led to an additional 0.80 QALY compared to T-DM1, resulting in an ICER of $153,950.19 per QALY gained. The PC strategies are considered more cost-effective than T-DM1 when the WTP threshold is set at $36,058.06 per QALY. However, this method is not cost effective for T-DXd. The probability of the PC strategies being cost-effective was 62%, whereas the probability of T-DXd was 0% when compared to T-DM1.

**Conclusion:**

PC is a cost-effective therapy for patients afflicted with HER-2-positive metastatic BC compared to T-DM1 from the perspective of China at a WTP threshold of $36,058.06 per QALY. Nevertheless, T-DXd is not as cost-effective as T-DM1, considering its current medication pricing. Therefore, reducing the cost of T-DXd could improve its overall cost-effectiveness.

## 1 Introduction

Breast cancer (BC) is a global health predicament that affects countless women annually, making it the leading cause of cancer-related deaths among women worldwide. (World Health Organization Breast cancer, 2024). Breast cancer is the second most common cancer worldwide, with 2.3 million new cases accounting for 11.6% of all new cancer cases, and is the fourth leading cause of cancer death (670,000 deaths, 6.9%) globally in 2022 (Global cancer burden growing, 2024). The prevalence and fatality rates of BC have persistently increased not only in developed nations, but also in developing regions (Global Burden of Disease Cancer, 2018). One distinct subtype of BC, human epidermal growth factor receptor-2 (HER-2)-positive metastatic breast cancer (MBC), is diagnosed in approximately 20% of women with BC (Tosello et al., 2018). The incidence of BC in China is among the highest worldwide (Qiu et al., 2021). Dissemination of early screening practices and refinement of treatment modalities will play pivotal roles in achieving this formidable and momentous objective. Neoadjuvant systemic therapy has become the standard treatment, using cytotoxic chemotherapy, endocrine therapy, and targeted therapy based on tumor biology (Teshome and Hunt, 2014; Harbeck and Gnant, 2017).

Overexpression of HER-2 is associated with unfavorable prognosis and shorter overall survival (OS). Nevertheless, the use of trastuzumab, a humanized monoclonal antibody targeting the extracellular domain of HER-2, in combination with chemotherapy has markedly enhanced the survival of HER-2 positive patients with BC (Slamon et al., 2001; Perez et al., 2014). For individuals with metastatic disease, the accepted initial treatment encompasses the administration of monoclonal antibodies, namely, trastuzumab and pertuzumab, along with chemotherapy. This therapeutic approach substantially extended the progression-free survival (PFS) to 18.7 months and OS to 57.1 months (Swain et al., 2020). If first-line trastuzumab treatment fails, second-line anti-HER-2 treatment is recommended. According to the 2023 National Comprehensive Cancer Network guidelines, the use of antibody-drug conjugates (ADCs), such as fam-trastuzumab deruxtecan-nxki (T-DXd) has been advocated (Gradishar et al., 2023). Based on the development and use of new anti-HER-2 drugs, HER-2-positive BC has shown the most significant improvement in survival, surpassing luminal BC. However, resistance to trastuzumab is inevitable, with 10% of patients experiencing primary drug resistance. To overcome drug resistance, drugs with different mechanisms of action must be replaced. Emerging therapeutic agents, including tyrosine kinase inhibitors (TKIs) and ADCs, have shown promise.

TKIs inhibit the cascade signaling pathways of the pan-HER family through their interaction with tyrosine kinases, thereby inhibiting the proliferation and metastasis of tumor cells (Butti et al., 2018; Ye et al., 2023). Pyrotinib, an irreversible pan-ErbB TKI, exhibits superior suppression of ErbB family receptors and promising antineoplastic activity compared with lapatinib, a reversible TKI (Ma et al., 2019). In the PHOEBE phase 3 study, 134 patients were randomly assigned to the pyrotinib plus capecitabine (PC) group and 133 to the lapatinib plus capecitabine (LC) group. The median follow-up period were 10.5 months in the PC group and 9.7 months for the LC group, respectively. The study demonstrated a significantly prolonged median PFS in the PC group compared to the LC group [12.5 months vs 6.8 months, hazard ratio (HR) 0.39, *p* < 0.0001] (Xu et al., 2021). Furthermore, OS also showed a benefit in the PC group, although the OS events of the two groups were not reached [NA vs. 26.9 months, HR 0.69, *p* = 0.02] with manageable toxicity (diarrhea and hand-foot syndrome) (Xu et al., 2022). Another investigation conducted at the National Cancer Center of China reported a comparable OS outcome of 59.9 months in the PC cohort based on a median follow-up duration of 69.3 months (Guan et al., 2023).

Trastuzumab emtansine (T-DM1) is a fusion of the HER-2 targeted antitumor efficacy of trastuzumab with potent cytotoxicity due to the microtubule-inhibitory agent, DM (Junttila et al., 2011). The EMILIA phase 3 study unveiled compelling evidence attesting to the unequivocal superiority of T-DM1 over LC in addressing the relentless advance of HER-2-positive MBC. Among the cohort of 991 patients subjected to randomization (T-DM1 = 495; LC = 496), and with an average follow-up duration of approximately 19 months, T-DM1 conferred a significantly protracted PFS when juxtaposed against LC (9.6 months vs 6.4 months, HR 0.65, *p* < 0.001), while the mantle of OS was also donned by T-DM1 (30.9 months vs 25.1 months, HR 0.68, *p* < 0.001) (Verma et al., 2012). Subsequent studies have shown that a longer median OS was observed with T-DM1 in comparison to LC (29.9 months vs 25.9 months, HR 0.75), adverse events (AEs) associated with thrombocytopenia and escalated serum aminotransferase levels (Dieras et al., 2017).

T-DXd is an antibody-drug conjugate consisting of a humanized anti-HER-2 monoclonal antibody linked to a topoisomerase I inhibitor payload through a tetrapeptide-based cleavable linker (Nakada et al., 2019). In the DESTINY-Breast 03 phase 3 trial, 524 patients were enrolled and randomly assigned to either the T-DXd (261) or T-DM1 (263) groups. The median duration of study follow-up was 28.4 months for T-DXd and 26.5 months for T-DM1. The median PFS was 28.8 months for T-DXd and 6.8 months for T-DM1 (HR 0.33, *p* < 0.0001). The median OS was not reached in either the T-DXd group or T-DM1 group [NR (40.5 months—NA) vs NR (34.0 months—NA), HR 0.64, *p* = 0.0037]. Treatment with T-DXd is associated with interstitial lung disease and pneumonitis in 10.5% of patients (Hurvitz et al., 2023).

Currently, no studies have compared the effectiveness of PC and ADCs as second-line anti-HER-2 treatments. To evaluate various treatments, in addition to clinical efficacy and safety, economic evaluation may play an important role in patients’ treatment choices. As a new treatment, T-DXd is costly. T-DM1 and pyrotinib are already covered by the Chinese medical insurance, and their prices will have some advantages. Therefore, to optimize the allocation of medical resources, it is necessary to evaluate their economic feasibility. In this context, we evaluated the cost-effectiveness of PC, T-DM1, and T-DXd in patients with HER-2-positive MBC who had previously been treated with trastuzumab from a Chinese payer perspective.

## 2 Materials and methods

A cost-effectiveness analysis (CEA) was used to guide decision making in this study. CEA relies on incremental analysis, which is an incremental analysis to compare the costs and outcomes of the intervention and comparator. If the intervention has lower costs and better outcomes than the comparator, it becomes the dominant treatment scheme. Conversely, if the intervention had higher costs and poorer outcomes than the comparator, it was considered inferior. When the intervention treatment has a higher cost and better outcome than the comparator, it is necessary to calculate the incremental cost-effectiveness ratio (ICER). The ICER represents the ratio of the cost difference to the outcome difference between the two schemes. An ICER value less than or equal to the predetermined threshold indicates that the intervention is more cost-effective than the comparator. If the ICER exceeded the threshold, the intervention was considered invalid (Gordon et al., 2020). To measure outcomes, the use of life years (LY) and quality-adjusted life years (QALY) is recommended. The QALY serves as an indicator of the impact of an intervention on a person’s overall quality of life. The formula for calculating ICER is as follows (Cai et al., 2019):

ICER = (Cost of Intervention—Cost of Comparator) / (Outcome of Intervention—Outcome of Comparator).

### 2.1 Network meta-analysis

We performed an extensive literature search using PubMed, Embase, and the Cochrane Central Register of Controlled Trials to identify relevant publications. The search was limited to manuscripts published before 1 August 2023. In addition, our search included ClinicalTrials. gov. Search terms used included, “Trastuzumab Deruxtecan,” “Trastuzumab Emtansine,” “pyrotinib plus capecitabine,” “lapatinib plus capecitabine,” “metastatic,” “advanced,” “breast cancer,” and “second-line” as medical subject keywords. Supplementary Figure S1 provides more details on the search filters used. To ensure the reliability of our analysis, we excluded duplicate reports from the same clinical trial, trials without a control group, trials involving other interventions, and nonrandomized trials.

For the statistical analysis, we utilized “meta” and “netmeta” software packages in R version 4.2.1 to conduct a Bayesian network meta-analysis. This allowed us to determine the hazard ratios (HRs) for OS and PFS when comparing trastuzumab-deruxtecan, T-DMI, and PC. The pooled HRs for PFS and OS were then used for the cost-effectiveness analysis.

To evaluate the risk of bias in the included clinical trials, we used the RevMan version 5.4. Employed a fixed-effects model because of limited data for assessing inter-trial heterogeneity (Rucker and Schwarzer, 2015).

### 2.2 Model structure

**FIGURE 1:**
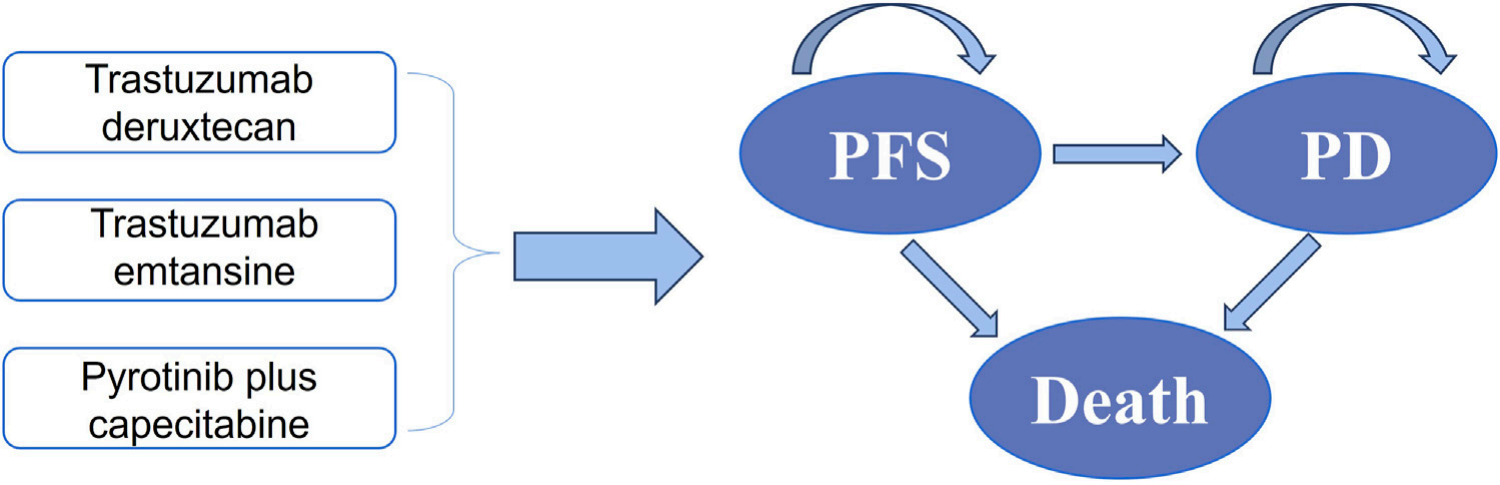
Partitioned survival model.

By establishing a partitioned survival model, Microsoft Excel was used to evaluate health-related costs and outcomes of different strategies for HER-2-positive MBC. PFS, progressive disease (PD), and death were incorporated into the model (Figure 1). This analysis primarily aimed to evaluate the cost-effectiveness of T-DXd and PC compared to T-DM1, which was used as a standard reference strategy. We set a 21-day cycle for10 years in the model. For both costs and utilities, a 5% discount was applied to account for the time value of money annually, and discount rates of 0% and 8% were explored in a sensitivity analysis (Gordon et al., 2020). For China, the willingness-to-pay (WTP) threshold was set at $36,058.06 (three times the GDP *per capita* in 2022) per QALY (Bertram et al., 2016). In the analysis, we assumed that PFS was the initial state and death was the absorbing state.

### 2.3 Clinical inputs

In the PC group, patients were administered a daily oral dose of 400 mg pyrotinib and twice daily oral dose of 1,000 mg/m^2^ capecitabine from days 1–14 of each 21-day cycle (Xu et al., 2021). Patients in the T-DXd and T-DM1 groups received either 5.4/kg of T-DXd and 3.6 mg/kg of T-DM1 via intravenous infusion every 3 weeks (Verma et al., 2012; Hurvitz et al., 2023). These treatment schemes were continued until the patient experienced disease progression, unacceptable toxicity, death, withdrawal of consent, the investigator’s decision, or completion of the study. High-resolution contrast-enhanced CT or MRI was performed for monitoring.

### 2.4 Efficacy estimates

To determine the drug efficacy, it is important to use the most reliable available evidence. For newer drugs, it is preferable to have clinical efficacy data from randomized controlled trials, if such data are available and applicable. In the EMILIA, DESTINY-Breast03, and PHOEBE trials, the primary endpoints were PFS and OS.

To analyze the survival data from the trials, the researchers used a software called GetData Graph Digitizer (version 2.26) to extract graphical data from the Kaplan–Meier (K-M) curves of both trials. Based on Guyot et al.’s approach (Guyot et al., 2012), individual patient data (IPD) were used to construct the parameter model among various distributions, such as gamma, Weibull, Gompertz, log-normal, log-logistic, exponential, and generalized gamma. Based on the Akaike information criterion (AIC) value, the researchers selected a parametric regression model.

Supplementary Figures S2–S4 provide the digitized K-M curves based on the data. The replicated KM OS and PFS curves of T-DM1 treatment were generated by pooling EMILIA, DESTINY-Breast03, and PHOEBE (Supplementary Figure S5). The IPD in the T-DM1 arm of the three trials was pooled and fitted with log-normal and generalized gamma distributions for PFS and OS, respectively. In addition, the researchers relied on the AIC statistic to objectively assess the kindness of fit of different distributions and ultimately selected the one that provided the best fit to their data. The final parametric model, including the selected distribution and its parameters, is presented in Supplementary Table S1. Supplementary Figure S6 illustrates the model-fitted K-M curves, which show how the survival probabilities change over time based on the selected parametric model.

### 2.5 Cost inputs

In this study, we only analyzed direct medical costs, including expenditures related to drugs, such as T-DXd, T-DM1, pyrotinib, and capecitabine (Chinese drug price of drug centralized bid procurement, 2023). Additionally, we considered expenses pertaining to diagnostic tests, severe AEs of grade 3 or 4, utilization of healthcare resources, treatments subsequent to disease progression, concomitant therapy cost, and end-of-life care (Table 1). The pharmaceutical costs were evaluated according to the bid-winning price (Chinese drug price of drug centralized bid procurement, 2023), and other cost data were obtained from the results of expert interviews. Table 2 shows the occurrence rates of notable grade 3 or 4 AEs observed with different treatments. To ascertain the appropriate medication dosage, we used the average weight and body surface area of a typical female patient in China, which are 59 kg and 1.61 m^2^, respectively (Report on the nutrition and chronic disease status of Chinese residents: people’s medical, 2020). In addition, for international comparisons, all expenditures are converted into United States dollars, using the exchange rate of July 2023 ($1 = ¥ 7.13).

**TABLE 1:** Input parameters of the model.

Parameter	Baseline value	Lower limit	Upper limit	Distribution	Source
Survival model of T-DM1					
Lognormal PFS survival model	meanlog = 2.1852	ND	ND	ND	Model fitting
	sdlog = 1.1752				
Generalised gamma OS survival model	mu = 3.6876	ND	ND	ND	Model fitting
	sigma = 0.8951				
	Q = 0.3992				
HR for OS (PC vs. T-DM1)	1.00	0.66	1.52	Lognormal	Network meta-analysis
HR for PFS (PC vs. T-DM1)	0.60	0.40	0.89	Lognormal	Network meta-analysis
HR for OS (T-DXd vs. T-DM1)	0.64	0.47	0.87	Lognormal	Network meta-analysis
HR for PFS (T-DXd vs. T-DM1)	0.33	0.26	0.43	Lognormal	Network meta-analysis
Drug cost (per cycle)					
T-DM1	1,084.54	867.64	1,301.45	Gamma	Chinese drug price of drug centralized bid procurement (2023)
Pyrotinib	1,051.18	840.94	1,261.41	Gamma	Chinese drug price of drug centralized bid procurement (2023)
Capecitabine	153.77	123.02	184.52	Gamma	Chinese drug price of drug centralized bid procurement (2023)
T-DXd	3,727.91	2982.33	4,473.49	Gamma	Chinese drug price of drug centralized bid procurement (2023)
Progressive disease therapy	2232.41	1785.93	2678.89	Gamma	Chinese drug price of drug centralized bid procurement (2023)
T-DM1	97.73	78.18	117.27	Gamma	Expert interviews
PC	103.37	82.69	124.04	Gamma	Expert interviews
T-DXd	175.66	140.53	210.79	Gamma	Expert interviews
Healthcare resource utilization cost					
PFS status (first cycle)	649.25	519.40	779.10	Gamma	Expert interviews
PFS status (follow-up cycle)	54.09	43.27	64.90	Gamma	Expert interviews
PD status	68.03	54.42	81.63	Gamma	Expert interviews
Concomitant therapy cost	21.32	17.05	25.58	Gamma	Expert interviews
End-of-life care	97.22	77.78	116.67	Gamma	Expert interviews
AEs cost					
Diarrhea	16.30	13.04	19.57	Gamma	Expert interviews
Vomiting	16.30	13.04	19.57	Gamma	Expert interviews
Neutropenia	123.00	98.40	147.60	Gamma	Expert interviews
Fatigue	22.91	18.33	27.49	Gamma	Expert interviews
Nausea	16.30	13.04	19.57	Gamma	Expert interviews
Anemia	79.10	63.28	94.92	Gamma	Expert interviews
Alanine aminotransferase increased	60.34	48.27	72.41	Gamma	Expert interviews
Aspartate aminotransferase increased	60.34	48.27	72.41	Gamma	Expert interviews
Health utility					
PFS state	0.850	0.680	1.000	Beta	Wu and Ma (2020)
PD state	0.520	0.420	0.620	Beta	Wu and Ma (2020)

Abbreviations: OS, overall survival; PFS, progression-free survival; PD, progressive disease; HRs, hazard ratios; AEs, adverse events; T-DM1, trastuzumab emtansine; T-DXd, trastuzumab deruxtecan; ND, not determined.

**TABLE 2:** The incidence of adverse events.

Grade ≥3 AEs	T-DM1	PC	T-DXd
Diarrhea	0.004	0.031	0.004
Vomiting	0.004	0.060	0.016
Neutropenia	0.031	0.060	0.058
Fatigue	0.008	0.010	0.051
Nausea	0.004	0.010	0.066
Anemia	0.042	0.010	0.058
Alanine aminotransferase increased	0.046	0.020	0.016
Aspartate aminotransferase increased	0.050	0.010	0.008

Abbreviations: AEs, adverse events; T-DM1, trastuzumab emtansine; PC, pyrotinib plus capecitabine; T-DXd, trastuzumab deruxtecan.

### 2.6 Utilities estimates

The utility score serves as a metric that appraises social functioning and overall health, encompassing physical, mental, and ailment-related facets. It is measured on a scale from 0 to 1, with 0 indicating the poorest health status or mortality and one indicating the optimal health status. In the context of MBC, the utility estimates for PFS and PD were determined to be 0.85 and 0.52, respectively (Wu and Ma, 2020) (Table 1).

### 2.7 Sensitivity analyses

We carefully reviewed the myriad factors listed in Table 1 to evaluate their influence on the sensitivity analysis results. These factors included costs, utilities, HRs (derived from a network meta-analysis), and probability.

To evaluate the robustness of the model results, we performed one-way deterministic sensitivity analyses by individually varying each input. If information was available, we used the reported 95% confidence interval (CI) to vary the model parameters. In cases where such information was not provided, we varied the parameters by ± 20% from the base case values (Table 1).

The 10,000 Monte Carlo simulations were used to analyze the probabilistic sensitivity. In each iteration, the model parameters are randomly sampled from a specified distribution. The parameters related to the HRs follow a log-normal distribution, whereas the cost parameters follow a gamma distribution. Variables, such as probability, HRs, and utility values were represented by a beta distribution.

The sensitivity analysis results were presented as cost-effectiveness acceptability curves (CEAC). This curve shows the probability that a given intervention is more cost-effective than an alternative intervention.

## 3 Results

### 3.1 Network meta-analysis

A network meta-analysis was conducted using a database search that identified 53 records. Among these, three phase III randomized clinical trials (EMILIA, DESTINY-Breast03, and PHOEBE) involving 1782 patients were included in the analysis. Supplementary Figure S7 provides a schematic model of the network meta-analysis. In the EMILIA trial, 991 patients underwent either LC (N = 496) or T-DM1 (N = 495). The DESTINY-Breast03 trial included 524 patients who received either T-DXd (N = 261) or T-DM1 (N = 263). The PHOEBE trial included 266 patients who were administered either PC (N = 134) or LC (N = 132). Supplementary Figure S8 shows the risk of bias. Based on the indirect comparisons made in the network meta-analysis, it is evident that both PC (HR 1.00, 95% CI, 0.66–1.52) and T-DXd (HR 0.64, 95% CI, 0.47–0.87) demonstrated significant improvements in OS compared to T-DM1-related survival. Moreover, the HRs for PFS were 0.60 (95% CI, 0.40–0.89) for PC and 0.33 (95% CI, 0.26–0.43) for T-DXd when compared to T-DM1 treatment.

### 3.2 Cost-effectiveness analysis

#### 3.2.1 Base-case analyses

Within the model encompassing 1782 patients, PC treatment yielded an additional 0.70 QALYs in comparison to T-DM1. This outcome translates into an ICER of $31,121.53 per QALY gained. Similarly, the administration of T-DXd treatment led to an additional 0.80 QALYs compared to T-DM1, resulting in an ICER of $153,950.19 per QALY gained (Table 3).

**TABLE 3:** Results of the base-case analysis.

Regimen	Total cost	LYs	QALYs	Incremental cost ($)	Incremental LYs	Incremental QALYs	ICER ($/QALY)
T-DM1	32,864	3.29	2.09				
PC	54,766	4.06	2.79	21,902	0.77	0.70	31,121.53
T-DXd	156,356	4.25	2.89	123,492	0.96	0.80	153,950.19

Abbreviations: LYs, life-years; QALYs, quality-adjusted life years; ICER, incremental cost-effectiveness ratio.

#### 3.2.2 Sensitivity analyses

**FIGURE 2:**
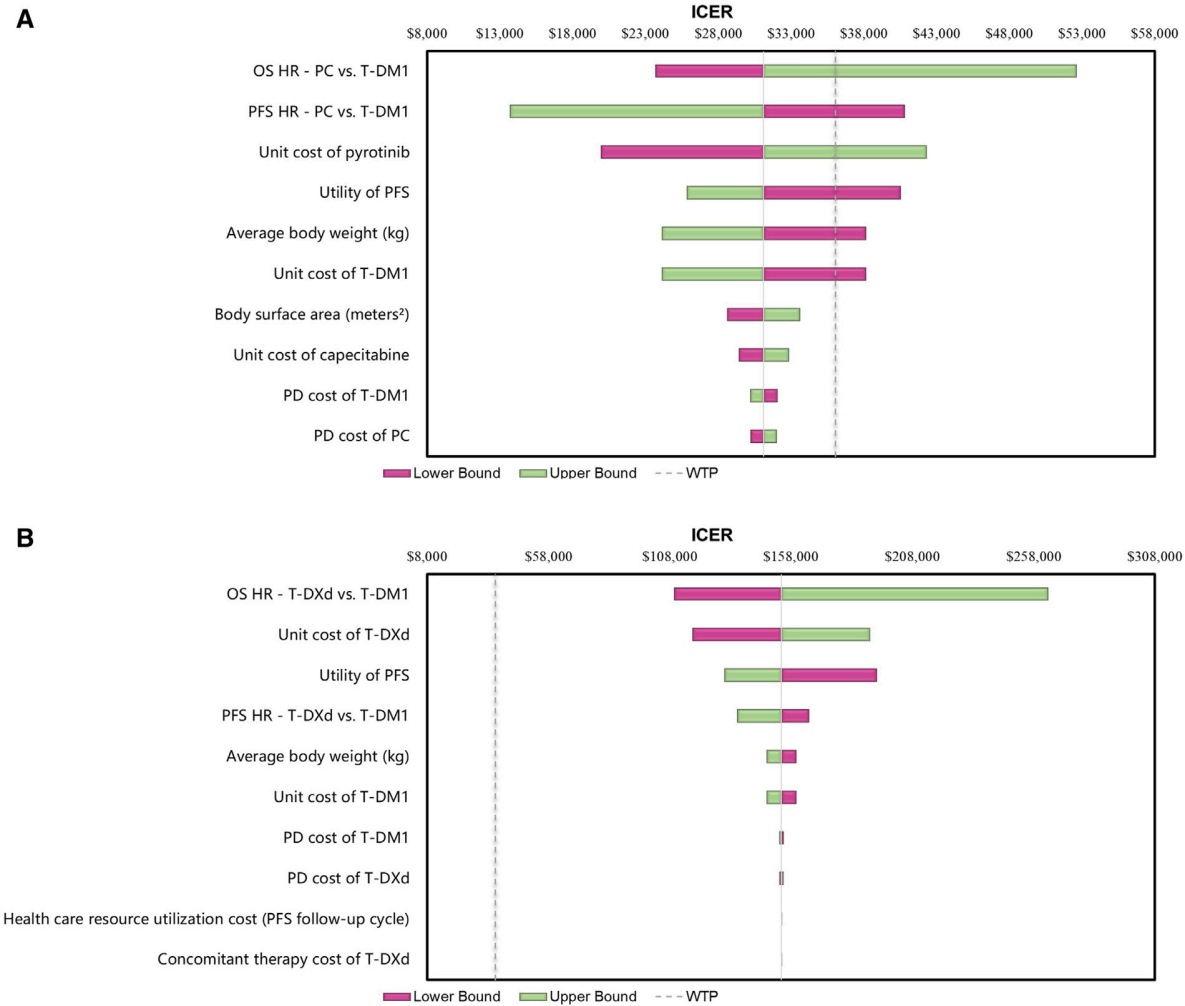
Tornado Diagrams of One-way Deterministic Sensitivity Analyses **(A)** One-way deterministic sensitivity analyses of PC in comparison with T-DM1. **(B)** One-way deterministic sensitivity analyses of T-DXd in comparison with T-DM1. Abbreviations: WTP, willingness to pay; OS, overall survival; PFS, progression-free survival; PD, progressive disease; HR, hazard ratio; ICER, incremental cost-effectiveness ratio; T-DM1, T-DMI; T-DXd, trastuzumab deruxtecan; PC, PC.

The results of the one-way sensitivity analyses indicated that the findings were highly influenced by the HRs of OS for the PC and T-DXd regimens compared with T-DM1 (Figure 2). Specifically, when comparing the PC regimen with T-DM1, the HRs of PFS and the cost of the pyrotinib regimen had a significant impact on ICERs. In contrast, when comparing T-DXd with T-DM1, the cost of T-DXd and the utility of PFS were found to be sensitive factors.

**FIGURE 3:**
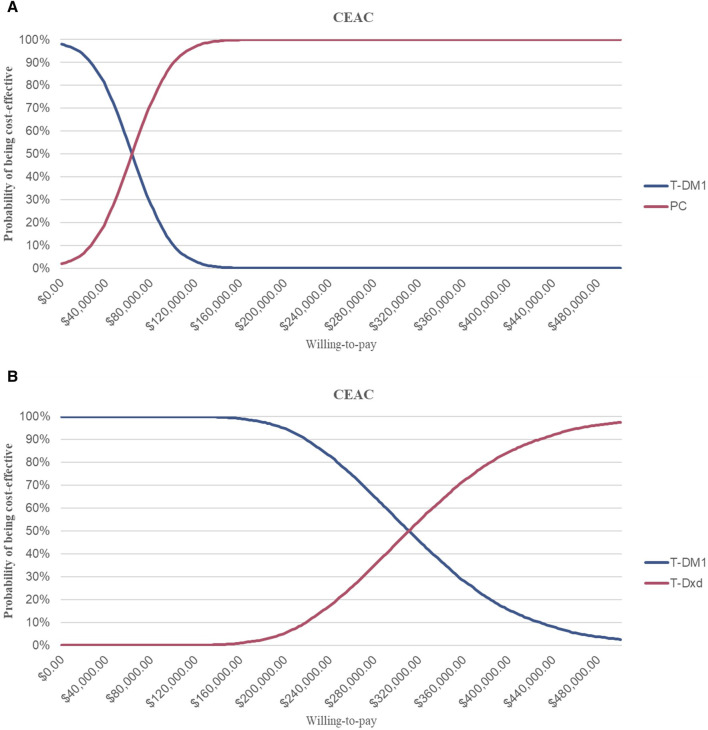
Cost-effectiveness Acceptability Curves **(A)** Cost-effectiveness acceptability curves of PC in comparison with T-DM1 **(B)** Cost-effectiveness acceptability curves of T-DXd in comparison with T-DM1. Abbreviations: CEAC, cost-effectiveness acceptability curve; T-DM1, T-DMI; T-DXd, trastuzumab deruximab; PC, PC.

**FIGURE 4:**
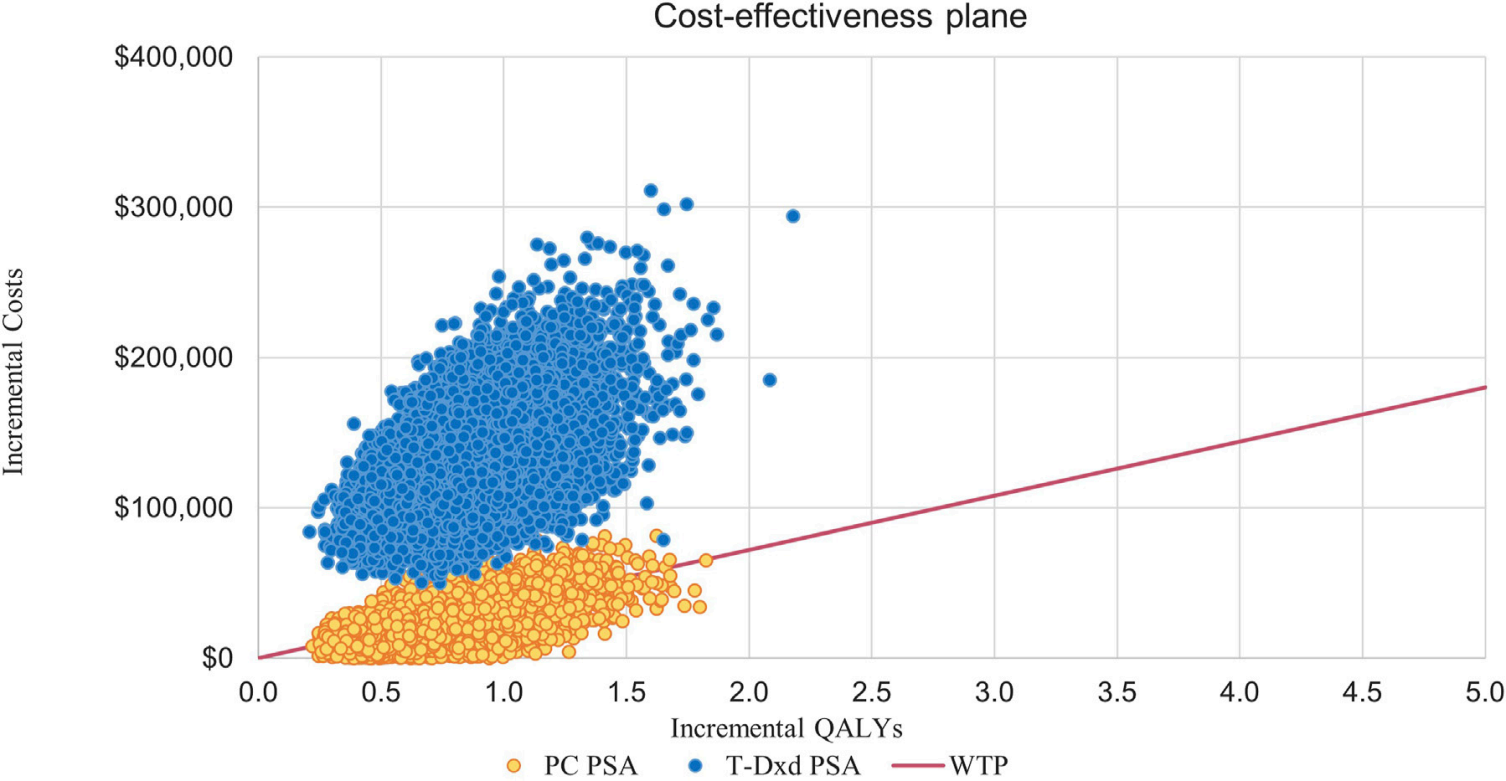
Incremental Cost-effectiveness Scatterplots. Abbreviations: T-DM1, T-DMI; T-DXd, trastuzumab deruxtecan; PC, PC; QALYs, quality-adjusted life-years; PSA, probabilistic sensitivity analysis; WTP, willingness to pay.

This suggests that PC strategies are more likely to be considered cost-effective compared to T- DM1 when the WTP threshold is set at $36,058.06 per QALY, with a 62% probability of cost-effectiveness. However, it is not cost-effective for T-DXd, for which the probability of T-DXd is 0% compared to that of T-DM1 (Figure 3; Figure 4).

#### 3.2.3 Scenario analysis

**FIGURE 5:**
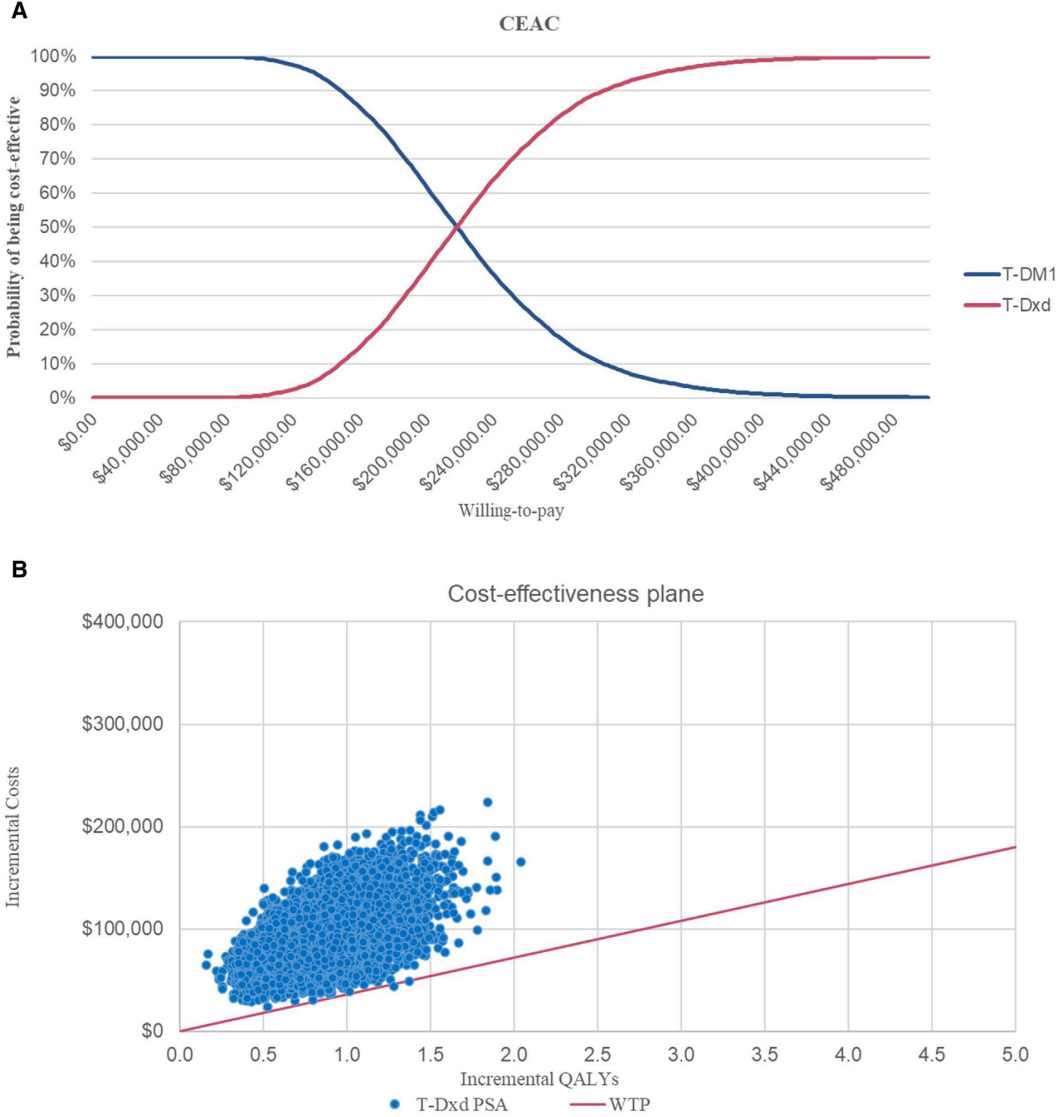
Cost-effectiveness Acceptability Curves and Incremental Cost-effectiveness Scatterplots of Scenario Analysis **(A)** Cost-effectiveness acceptability curves of T-DXd in comparison with T-DM1. **(B)** Incremental cost-effectiveness scatterplots of T-DXd in comparison with T-DM1. Abbreviations: WTP, willingness to pay; QALY, quality-adjusted life year; T-DM1, T-DMI; T-DXd, trastuzumab deruxtecan.

Owing to the high cost of T-DXd, it remains unattainable for numerous patients residing in China. Consequently, the T-DXd Patient Assistant Program was introduced to cater to the needs of Chinese patients seeking this medication. In the program, patients pay for three boxes of T-DXd and receive an additional box free of cost from Daiichi Sankyo Europe GmbH (the producer of T-DXd). In the scenario analysis, the utilization of T-DXd treatment resulted in an additional 0.80 QALYs compared to T-DM1, yielding an ICER of $108,216.39 per QALY (Table 4). The CEAC of the scenario analysis (Figure 5A) demonstrated that, despite a reduction in the ICER, the probability of T-DXd regimens being deemed cost-effective remained at 0% in comparison to T-DM1 at a WTP threshold of $36,058.06 per QALY. Figure 5B illustrates the scatterplot depicting incremental cost-effectiveness.

**TABLE 4:** Results of the scenario analysis.

Regimen	Total cost ($)	LYs	QALYs	Incremental cost ($)	Incremental LYs	Incremental QALYs	ICER ($/QALY)
TDM-1	32,864	3.29	2.09				
TDxd	119,670	4.25	2.89	86,806	0.96	0.80	108,216.39

Abbreviations: LYs, life-years; QALYs, quality-adjusted life years; ICER, incremental cost-effectiveness ratio.

## 4 Discussion

The results of the phase III DESTINY-Breast03 trial showed that the novel ADC T-DXd significantly improved PFS compared to T-DM1. Similarly, as an irreversible PAN-ErbB TKI, pyrotinib showed a more complete inhibitory effect on the ErbB family and exhibited good anti-tumor activity. They represent a major breakthrough in BC therapy, as they provide patients with more effective and promising options. In addition to these promising results, it is crucial to address the problem of high drug prices. New anticancer drugs, including T-DXd and pyrotinib, are often overpriced, which not only imposes a heavy financial burden on patients, but also puts pressure on national healthcare systems. As a result, the consumption of health resources soared, putting additional pressure on already limited resources. To ensure maximum and effective use of these scarce resources, an economic evaluation of new treatments and expensive drugs is necessary. This evaluation involves analyzing the cost-effectiveness of these treatments, considering factors, such as the incremental cost per additional unit of health benefit achieved. Moreover, economic evaluations can help guide pricing strategies for new drugs. Considering the cost-effectiveness of treatment, pharmaceutical companies can determine a fair and reasonable price that reflects the value they bring to patients and healthcare systems. This may help reduce the financial burden on patients and the healthcare system while still ensuring that drug developers can recoup their research and development costs.

Through cohort analysis of a patient cohort consisting of 1782 individuals, we established vital connections between PC, T-DXd, and T-DM1 via a network meta-analysis. These calculations revealed that PC treatment, when compared with T-DM1, yielded an additional 0.70 QALYs, ultimately culminating in an ICER of $31,121.53 per QALY. In a similar vein, T-DXd yielded an additional 0.80 QALYs compared to T-DM1, resulting in an ICER of $153,950.19 per QALY. Based on the given WTP threshold of $36,058.06 per QALY, a comprehensive analysis showed that there was a 62% probability that the PC strategies would be considered cost-effective. This implies that PC strategies, when compared with T-DM1, demonstrate a higher likelihood of providing value for the expenditure incurred. With the notable fluctuations in pyrotinib’s price pre and post price negotiations, it can be seen that it is more important to include it in the patient’s National Reimbursement Drug List (NRDL). Notably, in some developed regions, such as the United States and the European Union, pyrotinib is not commercially available. Thus, to a certain extent, this study provides a theoretical groundwork and compass to expedite the global commercialization of domestic pharmaceutical products. T-DXd has not yet been included in the NRDL, and should it be, the price is anticipated to undergo a substantial reduction, thereby granting a wider array of options to patients with cancer.

Using the database for literature search, we found 11 studies that examined the cost-effectiveness of T-DXd (Supplementary Table S2). Specifically, four studies assessed the cost-effectiveness of T-DXd and T-DM1 in HER-2-positive MBC, whereas seven studies investigated the cost-effectiveness of T-DXd compared with chemotherapy in HER-2-low advanced or MBC. The findings from most of these studies indicate that despite its outstanding efficacy, T-DXd is not considered cost-effective in many countries because of its high cost. Given the background of China’s healthcare system, it is estimated that a price reduction of at least 65% should be considered for T-DXd to achieve cost-effectiveness. This price reduction may make T-DXd more acceptable to many people, thereby increasing its overall use and potential health benefits. Pyrotinib is covered by the Chinese medical insurance, and our findings indicate that it is considered cost-effective when the threshold is set at three times the *per capita* GDP. However, the high cost of tumor treatment, including various medical expenses, remains a substantial barrier for many patients.

Excessive use of cancer drugs, often referred to as economic toxicity, is a huge challenge in both rich and emerging economies (Smith et al., 2022). Therefore, patients and the healthcare system bear a heavy economic burden, which can ultimately result in poor prognosis for patients or even cause them to abandon their treatment. Ensuring that patients have access to innovative drugs is critical, as it is as important as solving the problem of economic toxicity (Gyawali, 2017). In the United States, a lack of transparency and strong federal oversight have substantially contributed to the high price of medicines (Prasad et al., 2017). This lack of regulation allows drug companies to set prices at levels that patients and healthcare systems often cannot afford. Consequently, the United States has some of the highest drug costs worldwide. However, China’s State Council recognizes the importance of economic evaluation in multilateral negotiations and has implemented a priority policy for innovative drugs based on pharmacoeconomic (Wan et al., 2017; Liao et al., 2019). Considering the economic value of these drugs, China aims to provide objective data that can serve as a reference for the formulation of a universal health insurance. This practice also helps guide a more efficient and rational allocation of medical resources. Overall, addressing the high prices of anticancer drugs is crucial in both high- and middle-income countries. It affects not only the financial wellbeing of patients, but also the outcome of their treatment. Moreover, given the dynamic nature of healthcare systems, some potential external factors that could also influence the cost-effectiveness outcomes over the 10-year time horizon, such as changes in healthcare policies, drug pricing, and technological advancements.

### 4.1 Limitations

First, the long-term projections of PFS and OS in this study were related to uncertainty. It is challenging to accurately predict the results after the duration of clinical trials. In addition, the OS curves for PHOEBE and DESTINY-Breast 03 were immature. In real-world clinical practice, individual differences among patients may significantly affect the long-term outcomes. Therefore, the inclusion of more data from real-world studies may help validate the model and improve the accuracy of predictions of long-term results. Second, this study did not consider the costs associated with grade 1 or 2 AEs. This may lead to an underestimation of the total cost of treatment with PC, T-DXd, and T-DM1. However, the study found that the results were not sensitive to parameters related to AEs, suggesting that the exclusion of grade 1 or 2 AE costs did not significantly affect costs. Third, the utility values used in this study were obtained from previously published studies. However, utility values may not be aimed at the Chinese, which means that they may not be directly applicable to a Chinese background. Fourth, the results should be interpreted carefully when applied to areas other than China. This is because pyrotinib is only approved for use in China. Finally, in the course of the modeling process, direct medical cost data were obtained from expert interviews, which may have been subject to potential biases arising from regional disparities.

## 5 Conclusion

In conclusion, PC is a cost-effective therapy for patients afflicted with HER-2-positive MBC compared to T-DM1 from the perspective of China, at a WTP threshold of $36,058.06 per QALY. Nevertheless, T-DXd exhibits diminished cost-effectiveness compared to T-DM1, considering its current medication pricing. Thus, reducing the cost of T-DXd may enhance its overall cost-effectiveness.

## Data Availability

The original contributions presented in the study are included in the article/Supplementary Material, further inquiries can be directed to the corresponding authors.
