# Corrigendum: The value of second-line anti-HER2 therapy in metastatic HER-2 positive patients: a cost-effectiveness analysis in China

**DOI:** 10.3389/fphar.2024.1519505

**Published:** 2024-11-11

**Authors:** Lu Li, Shilei Yang, Fengqi Fang, Li Tian, Ying He, Jia Li, Yanwei Chen, Deshi Dong

**Affiliations:** ^1^ Department of Pharmacy, First Affiliated Hospital of Dalian Medical University, Dalian, China; ^2^ Department of Oncology, First Affiliated Hospital of Dalian Medical University, Dalian, China

**Keywords:** HER-2-positive metastatic breast cancer, network meta-analysis, cost-effectiveness analysis, pyrotinib plus capecitabine, T-DM1, T-DXd

In the published article, there was an error. T-DXd was indicated for comparison with PC strategies instead of T-DM1.

A correction has been made to the **Abstract**, *Findings.* This sentence previously stated:

“The PC strategies are considered more cost-effective than T-DXd when the WTP threshold is set at $36,058.06 per QALY.”

The corrected sentence appears below:

“The PC strategies are considered more cost-effective than T-DM1 when the WTP threshold is set at $36,058.06 per QALY.”

The authors apologize for this error and state that this does not change the scientific conclusions of the article in any way. The original article has been updated.

