# Supplementary material for: The value of second-line anti-HER2 therapy in metastatic HER-2 positive patients: a cost-effectiveness analysis in China
Source: Front Pharmacol. 2024 Jul 12;15:1382120. doi: 10.3389/fphar.2024.1382120 (PMC11282487; doi:10.3389/fphar.2024.1382120)
Supplement: Supplementary file 1 [file Table1.DOCX]

Supplementary Material

**1 Supplementary Figures and Tables**

- 1. **Supplementary Figures**

**
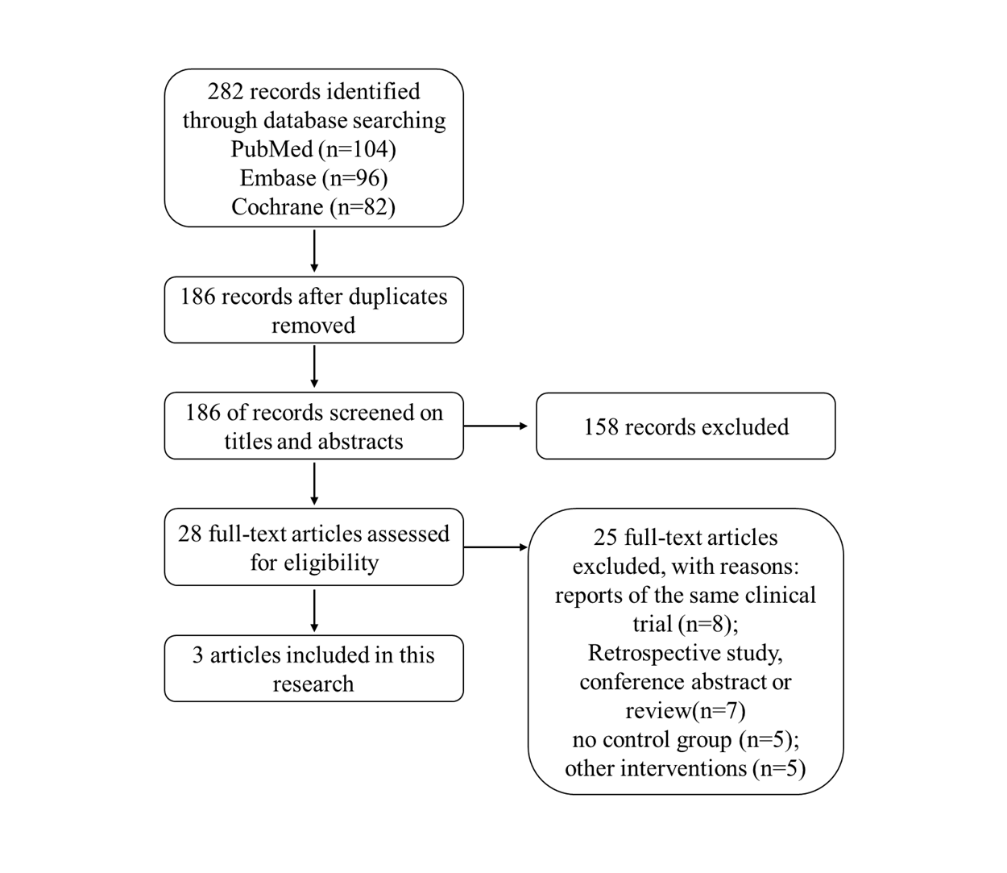
**

**Supplementary Figure 1** Flowchart of Study Selection


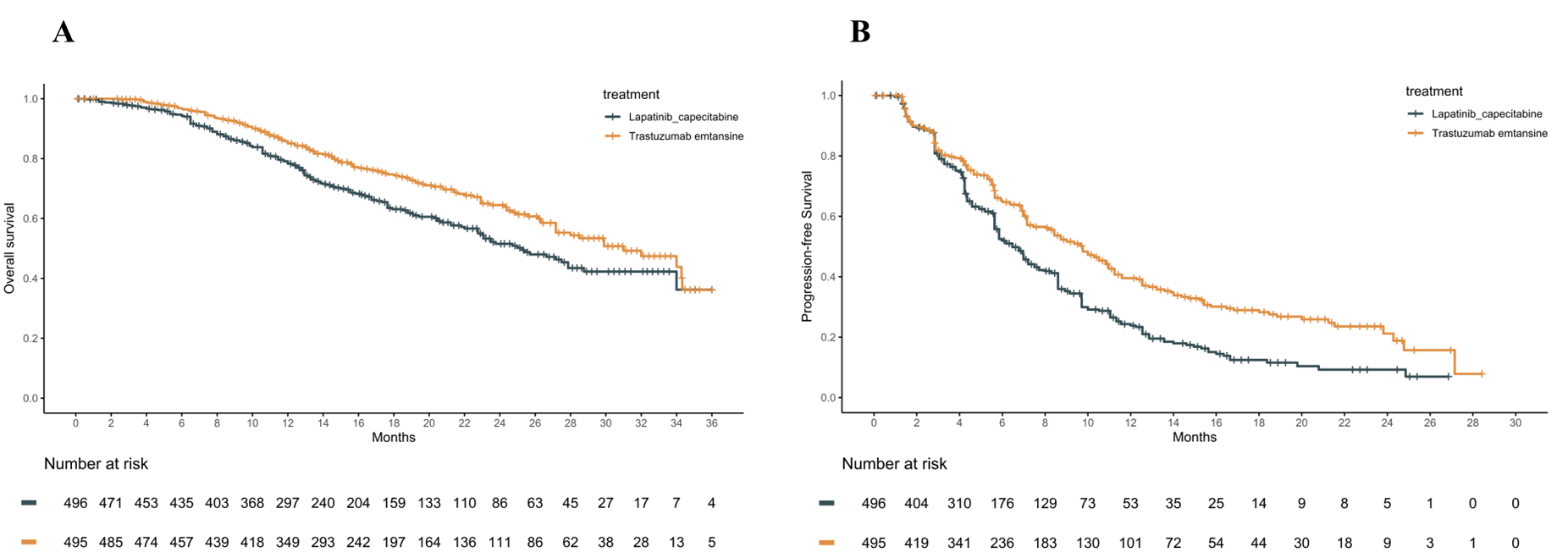


**Supplementary Figure 2** The replicated KM OS (A) and PFS (B) curves of Trastuzumab emtansine (orange) and lapatinib plus capecitabine treatments (blue) in EMILIA trial.

Abbreviations: OS, overall survival; PFS, progression-free survival; KM, Kaplan-Meier.


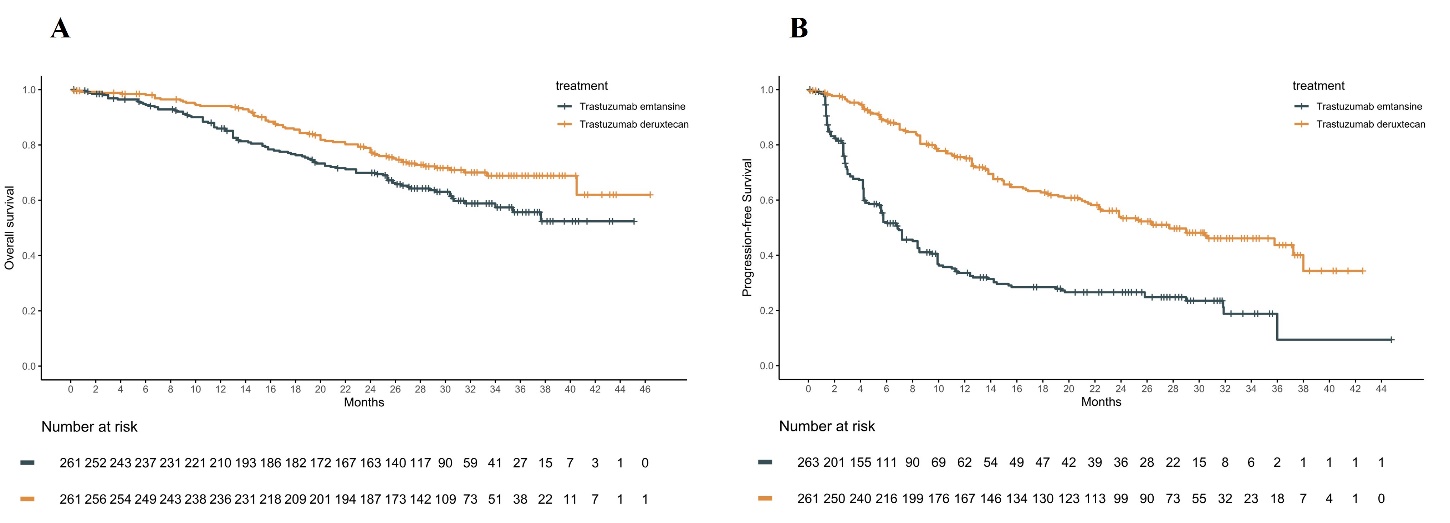


**Supplementary Figure 3** The replicated KM OS (A) and PFS (B) curves of Trastuzumab deruxtecan (orange) and Trastuzumab emtansine treatments (blue) in DESTINY-Breast03 trial.

Abbreviations: OS, overall survival; PFS, progression-free survival; KM, Kaplan-Meier.


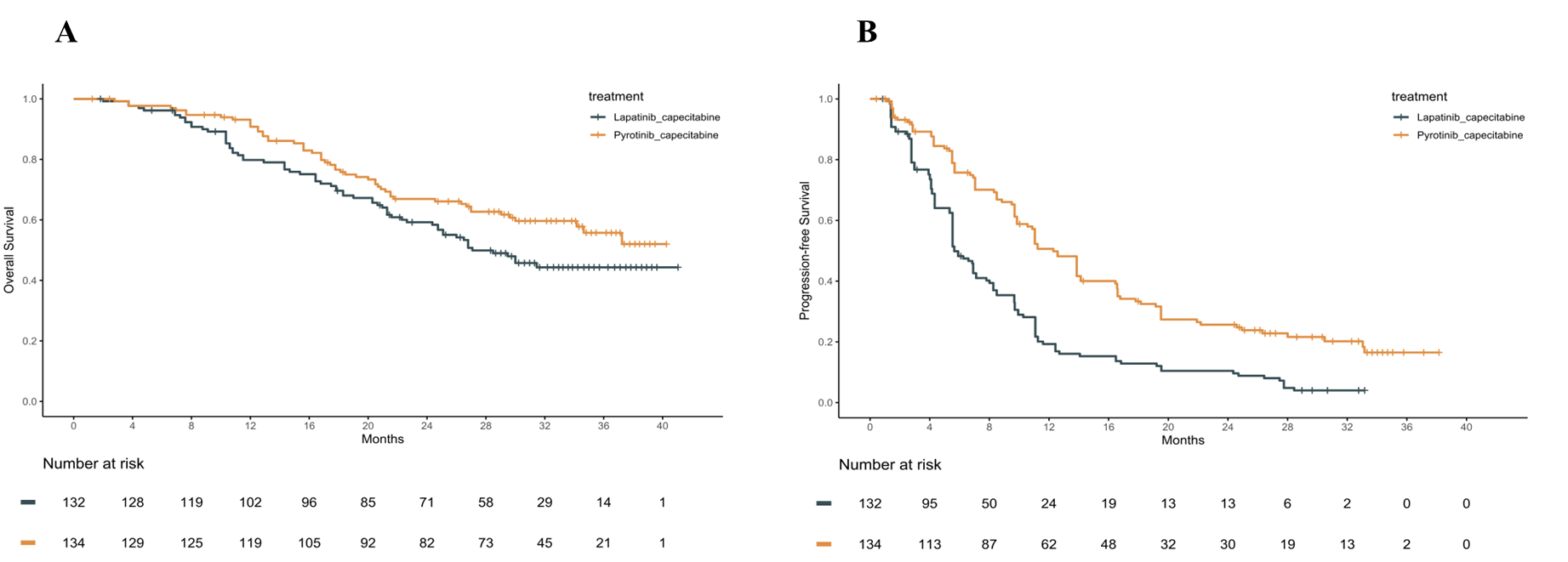


**Supplementary Figure 4** The replicated KM PFS curves of pyrotinib plus capecitabine (orange) and lapatinib plus capecitabine (blue) in PHOEBE trial.

Abbreviations: OS, overall survival; PFS, progression-free survival; KM, Kaplan-Meier.

**
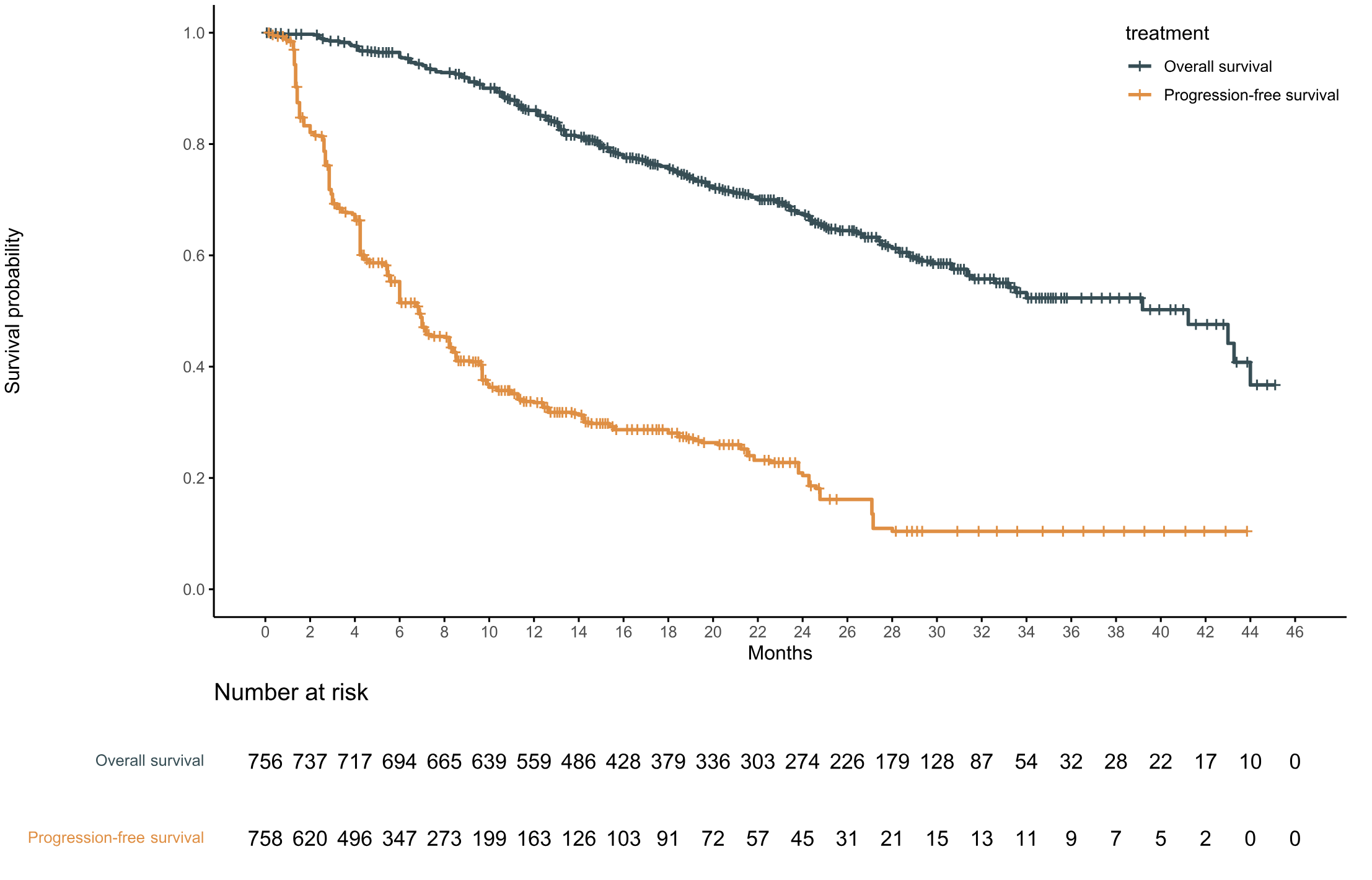
**

**Supplementary Figure 5** The replicated KM OS (blue) and PFS (orange) curves of trastuzumab emtansine treatment by pooling the EMILIA , PHOEBE, and DESTINY-Breast03 trials.

Abbreviations: OS, overall survival; PFS, progression-free survival; KM, Kaplan-Meier.


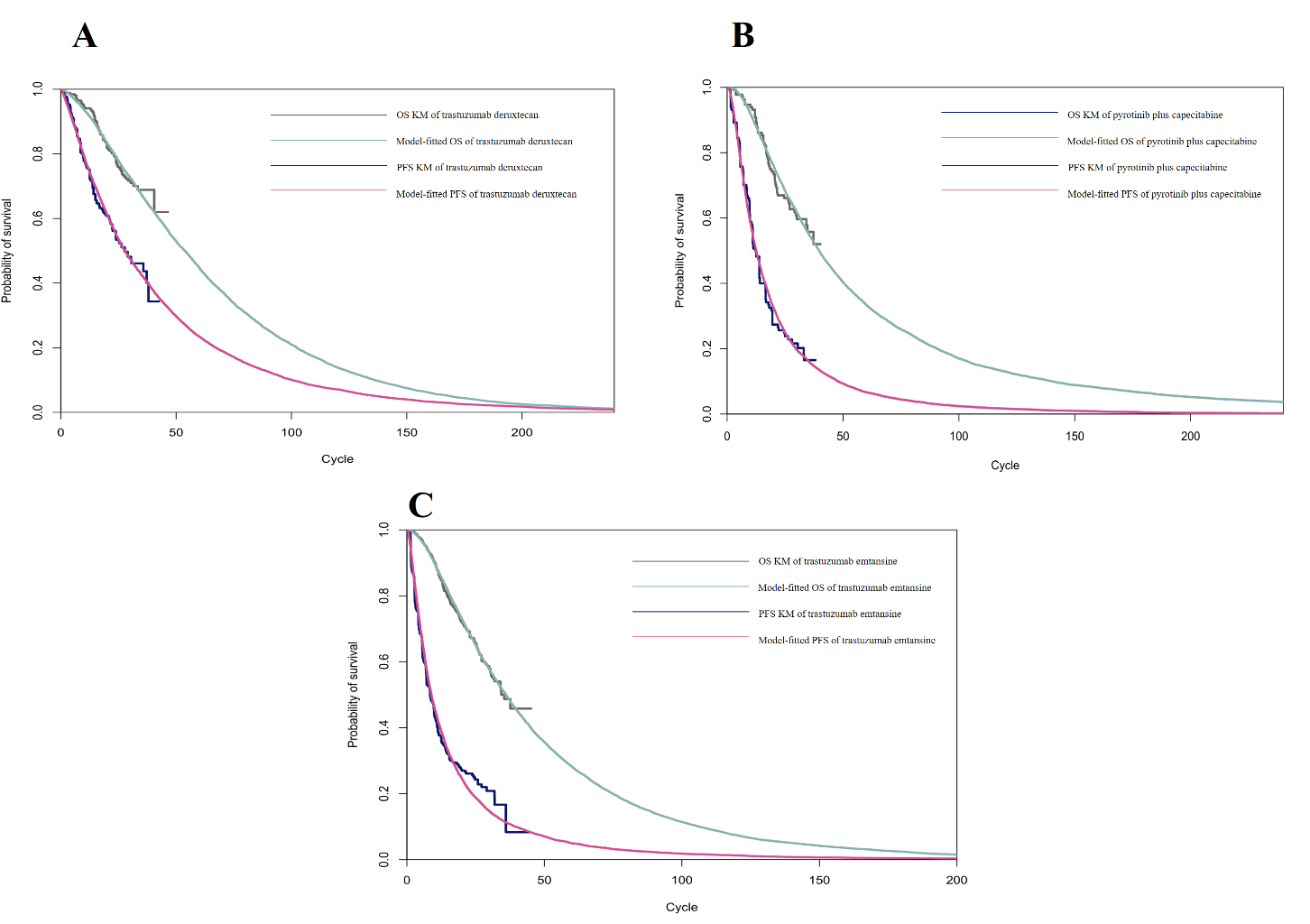


**Supplementary Figure 6** Original versus model-fitted Kaplan-Meier curves. The solid green lines represent the modeled survival curves of the OS of the three regimens, the solid pink lines represent the modeled survival curves of the PFS of the three regimens and the gray and blue lines are the actual survival curves. (A) Actual and modeled OS and PFS curve of trastuzumab deruxtecan; (B) Actual and modeled PFS curve of pyrotinib plus capecitabine. (C) Actual and modeled OS and PFS curve of trastuzumab emtansine by pooling the EMILIA and DESTINY-Breast03 trials. Each cycle of the x-axis is one month.

Abbreviations: OS, overall survival; PFS, progression-free survival; KM, Kaplan-Meier.

**
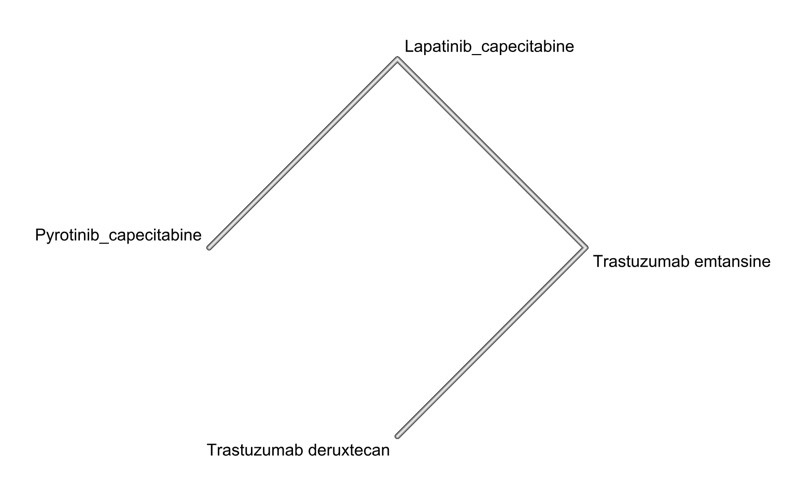
**

**Supplementary Figure 7** Model Schematic for Network Meta-analysis

**
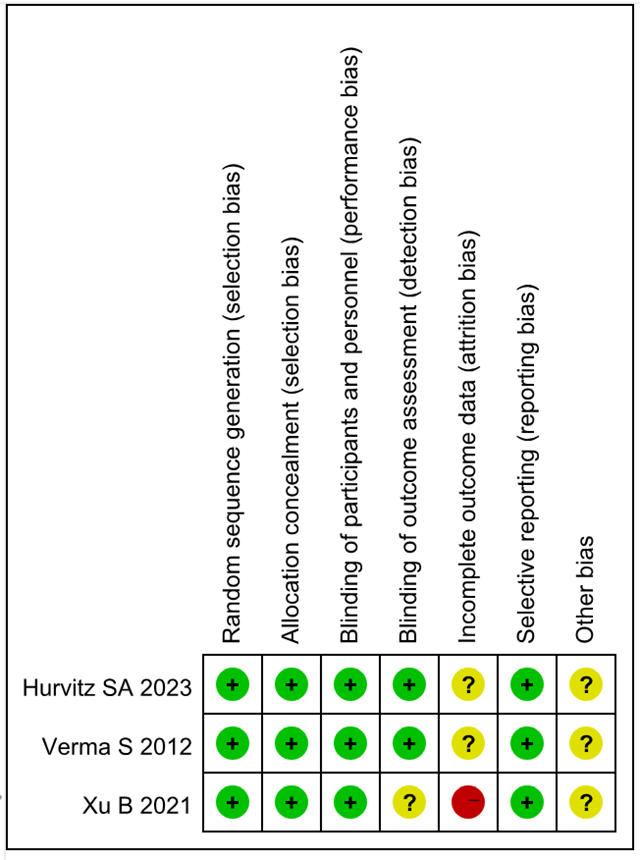
**

**Supplementary Figure 8** Risk of Bias Summary

- 1. **Supplementary Tables**

**Supplementary table 1** Parameters of parametric models for virtual time-to-event data.

| Trial names | Treatment regimens | Endpoint | Distribution | Distribution information | AIC |
| --- | --- | --- | --- | --- | --- |
| EMILIA | T-DM1 | OS | Log-normal | meanlog:3.4419 sdlog:0.8741 | 1422.860 |
|  |  | PFS | Generalised gamma | mu:1.9728 sigma:1.1188  Q:-0.5920 | 1857.279 |
|  | LC | OS | Log-logistic | shape:1.816 scale:24.871 | 1654.613 |
|  |  | PFS | Log-normal | meanlog:1.8855 sdlog:0.8481 | 1881.777 |
| DESTINY-Breast03 | T-Dxd | OS | Log-logistic | shape:1.58 scale:55.77 | 795.1248 |
|  |  | PFS | Log-logistic | shape:1.345 scale:27.544 | 1103.086 |
|  | T-DM1 | OS | Log-logistic | shape:1.436 scale:42.248 | 979.4388 |
|  |  | PFS | Generalised gamma | mu:1.4548 sigma:1.2783  Q:-1.397 | 1152.353 |
| PHOEBE | PC | OS | Log-normal | meanlog:3.689 sdlog:0.984 | 536.5060 |
|  |  | PFS | Log-normal | meanlog:2.5212 sdlog:1.0416 | 770.8800 |
|  | LC | OS | Log-normal | meanlog:3.3839 sdlog:0.9561 | 640.7456 |
|  |  | PFS | Log-normal | meanlog:1.8586 sdlog:0.8700 | 758.2841 |
| Pooling data of EMILIA and DESTINY-Breast03 | T-DM1 | OS | Log-logistic | shape:1.7521 scale:34.8555 | 2416.257 |
|  |  | PFS | Generalised gamma | mu:1.9728 sigma:1.1188  Q:-0.5920 | 3024.882 |
| Abbreviations: AIC, Akaike information criterion; OS, Overall survival; PFS, Progression-free survival; T-DM1, trastuzumab emtansine; LC, lapatinib plus capecitabine; T-Dxd, trastuzumab deruxtecan; PC, Pyrotinib plus capecitabine. | | | | | |

**Supplementary table 2** Summary of cost-effectiveness analyses for T-DXd in HER-2 positive breast cancer

| Study | Treatment | Perspective | Disease | Model | WTP threshold  (per QALY) | ICER |
| --- | --- | --- | --- | --- | --- | --- |
| Lang, Y 2022^1^ | T-DXd;  Chemotherapy | The United States payer | HER2-low advanced or metastatic breast cancer | Partitioned survival model | $150,000 | All patients group: $346,571.8/QALY;  HR-positive subgroup: $337,789.4/QALY |
| Yang, J 2023^2^ | T-DXd;  Chemotherapy | The United States payer | HER2-low metastatic breast cancer | Markov model | $150,000 | Overall HER2-low population: $317,494/QALY;  HR-positive subgroup: $353,903/QALY;  HR-negative subgroup: $259,825/QALY |
| Huang, X 2023^3^ | T-DXd;  Physician's choice of chemotherapy | The United States payers;  The Chinese healthcare system | Metastatic breast cancer with low HER2 expression | Markov model | US: $150,000  China: $36,475 | US:  $259,452.05/ QALY  China: $87,646.40/QALY |
| Zhu, Y 2022^4^ | T-DXd;  Chemotherapy | The United States payer | HER2-low advanced breast cancer | Markov model | $150,000 | Overall HER2-low patients:  $296,873/QALY  HR-positive subgroup: $318,944/QALY  HR- negative subgroup: $197,355/QALY |
| Zhan, M 2023^5^ | T-DXd;  Physician's choice of chemotherapy | Chinese healthcare system | HER2-low advanced breast cancer | Partitioned survival model | $357,96.83 | Overall HER2-low patients:  $336,026.77/QALY  HR-positive subgroup: $274,905.72/QALY |
| Shi, D 2023^6^ | T-DXd; Chemotherapy | A third-party payer in the United States | HER2-low advanced breast cancer | Partitioned survival model | $100,000 | All HER2-low advanced BC patients: $83,892/QALY  HER2+ advanced BC patients:  $82,808/QALY  HER2- advanced BC patients:  $93,358/QALY |
| Peng, Y 2023^7^ | T-DXd; Physician's choice of chemotherapy | The US payer perspective | HER2-Low advanced breast cancer | Partitioned survival model | $200,000 | Overall HER2-low patients:  $307,751/QALY  HR-positive subgroup: $383,776 /QALY  HR- negative subgroup: $194,424 /QALY |
| Zhu, Y 2022^8^ | T-DXd;  T-DM1 | The US payer;  The Chinese payer | HER2-positive metastatic breast cancer | Markov model | US: 150,000  China: 37,653 | US: $13,342/QALY  China: $186,017/QALY |
| Wang, J 2022^9^ | T-DXd;  T-DM1 | The US payer | HER2-positive metastatic breast cancer | Markov model | $50,000; $100,000; $150,000; $200,000 | $220,533/QALY |
| Paulissen, J 2023^10^ | T-DXd;  T-DM1 | Finnish payer’s perspective | HER2-positive unresectable and/or metastatic breast cancer | Partitioned survival model | €72,000; €139,000 | €55,360/QALY |
| Yang, J 2022^11^ | T-DXd;  T-DM1 | The US medical system;  The Chinese medical system | HER2-positive metastatic breast cancer | Markov model | US: $150,000  China: $37,653 | US: $ 82,112/QALY  China: $305,041/QALY |

**References**

1. Lang Y, Wu B, Liu X. Economic Evaluation of Trastuzumab Deruxtecan in Previously Treated HER2-Low Advanced Breast Cancer in the United States. *Breast Cancer (Dove Med Press)*. 2022;14:453-463. doi:10.2147/bctt.S389696

2. Yang J, Han J, Zeng N, Yan X. Cost-effectiveness of trastuzumab deruxtecan in previously treated human epidermal growth factor receptor 2-low metastatic breast cancer. *Ther Adv Med Oncol*. 2023;15:17588359231169983. doi:10.1177/17588359231169983

3. Huang X, Lin D, Lin S, et al. Cost-effectiveness and Value-based Pricing of Trastuzumab Deruxtecan in Metastatic Breast Cancer With Low HER2 Expression. *Clin Breast Cancer*. Jul 2023;23(5):508-518. doi:10.1016/j.clbc.2023.03.013

4. Zhu Y, Liu K, Zhu X, Qin Q, Zhu H. Trastuzumab deruxtecan versus chemotherapy for patients with HER2-low advanced breast cancer: A US-based cost-effectiveness analysis. *Front Pharmacol*. 2022;13:1025243. doi:10.3389/fphar.2022.1025243

5. Zhan M, Huang Z, Xu T, Xu X, Zheng H, Wu F. Cost-effectiveness analysis of trastuzumab deruxtecan in patients with HER2-low advanced breast cancer based on DESTINY-Breast04. *Front Public Health*. 2023;11:1049947. doi:10.3389/fpubh.2023.1049947

6. Shi D, Liang X, Li Y, Chen L. Cost-effectiveness of trastuzumab deruxtecan for previously treated HER2-low advanced breast cancer. *PLoS One*. 2023;18(8):e0290507. doi:10.1371/journal.pone.0290507

7. Peng Y, Wang L, Peng L, et al. Cost-Effectiveness of Trastuzumab Deruxtecan for HER2-Low Advanced Breast Cancer in the United States. *Clin Ther*. Aug 1 2023;doi:10.1016/j.clinthera.2023.07.014

8. Zhu Y, Liu K, Wang M, Wang K, Zhu H. Trastuzumab deruxtecan versus trastuzumab emtansine for patients with human epidermal growth factor receptor 2-positive metastatic breast cancer: A cost-effectiveness analysis. *Breast*. Dec 2022;66:191-198. doi:10.1016/j.breast.2022.10.010

9. Wang J, Yi Y, Wan X, Zeng X, Peng Y, Tan C. Cost-Effectiveness Analysis of Trastuzumab Deruxtecan versus Trastuzumab Emtansine in Human Epidermal Growth Factor Receptor 2-Positive Metastatic Breast Cancer in the USA. *Adv Ther*. Oct 2022;39(10):4583-4593. doi:10.1007/s12325-022-02273-4

10. Paulissen JHJ, Seddik AH, Dunton KJ, et al. Cost-effectiveness model of trastuzumab deruxtecan as second-line treatment in HER2-positive unresectable and/or metastatic breast cancer in Finland. *Eur J Health Econ*. Jul 24 2023;doi:10.1007/s10198-023-01617-3

11. Yang J, Han J, Zhang Y, Muhetaer M, Chen N, Yan X. Cost-effectiveness analysis of trastuzumab deruxtecan versus trastuzumab emtansine for HER2-positive breast cancer. *Front Pharmacol*. 2022;13:924126. doi:10.3389/fphar.2022.924126
